# Secretory prostate apoptosis response (Par)-4 sensitizes multicellular spheroids (MCS) of glioblastoma multiforme cells to tamoxifen-induced cell death

**DOI:** 10.1016/j.fob.2014.11.005

**Published:** 2014-11-21

**Authors:** Jayashree C. Jagtap, D. Parveen, Reecha D. Shah, Aarti Desai, Dipali Bhosale, Ashish Chugh, Deepak Ranade, Swapnil Karnik, Bhushan Khedkar, Aaishwarya Mathur, Kumar Natesh, Goparaju Chandrika, Padma Shastry

**Affiliations:** a National Centre for Cell Science (NCCS), Pune, India; b Persistent Systems Ltd., Pune, India; c Department of Neurosurgery, Cimet’s Inamdar Multispeciality Hospital, Pune, India; d Department of Neurosurgery, D.Y. Patil Medical College, Pune, India; e Department of Histopathology, Ruby Hall Clinic, Pune, India

**Keywords:** CM, conditioned medium, GBM, glioblastoma multiforme, LGG, low grade gliomas, MCS, multicellular spheroids, TAM, tamoxifen, TCGA, The Cancer Genome Atlas, Glioblastoma, Par-4, Drug resistance, Multicellular spheroids (MCS), Tamoxifen, Cytotoxicity

## Abstract

Glioblastoma multiforme (GBM) is the most malignant form of brain tumor and is associated with resistance to conventional therapy and poor patient survival. Prostate apoptosis response (Par)-4, a tumor suppressor, is expressed as both an intracellular and secretory/extracellular protein. Though secretory Par-4 induces apoptosis in cancer cells, its potential in drug-resistant tumors remains to be fully explored. Multicellular spheroids (MCS) of cancer cells often acquire multi-drug resistance and serve as ideal experimental models. We investigated the role of Par-4 in Tamoxifen (TAM)-induced cell death in MCS of human cell lines and primary cultures of GBM tumors. TCGA and REMBRANT data analysis revealed that low levels of Par-4 correlated with low survival period (21.85 ± 19.30 days) in GBM but not in astrocytomas (59.13 ± 47.26 days) and oligodendrogliomas (58.04 ± 59.80 days) suggesting low PAWR expression as a predictive risk factor in GBM. Consistently, MCS of human cell lines and primary cultures displayed low Par-4 expression, high level of chemo-resistance genes and were resistant to TAM-induced cytotoxicity. In monolayer cells, TAM-induced cytotoxicity was associated with enhanced expression of Par-4 and was alleviated by silencing of Par-4 using specific siRNA. TAM effectively induced secretory Par-4 in conditioned medium (CM) of cells cultured as monolayer but not in MCS. Moreover, MCS were rendered sensitive to TAM-induced cell death by exposure to conditioned medium (CM)-containing Par-4 (derived from TAM-treated monolayer cells). Also TAM reduced the expression of Akt and PKCζ in GBM cells cultured as monolayer but not in MCS. Importantly, combination of TAM with inhibitors to PI3K inhibitor (LY294002) or PKCζ resulted in secretion of Par-4 and cell death in MCS. Since membrane GRP78 is overexpressed in most cancer cells but not normal cells, and secretory Par-4 induces apoptosis by binding to membrane GRP78, secretory Par-4 is an attractive candidate for potentially overcoming therapy-resistance not only in malignant glioma but in broad spectrum of cancers.

## Highlights

• Multicellular spheroids (MCS) express low levels of Par-4 and are resistant to TAM-induced cytotoxicity.
• Par-4 is secreted in TAM-treated monolayers but not MCS and is crucial for cell death.
• Secretory Par-4 (from TAM-treated cells) renders MCS sensitive to cell death.
• Inhibitors to AKT/PKCζ sensitized MCS to TAM-induced cytotoxicity.
• A combination of TAM with PI3K inhibitor resulted in secretory Par-4.

## 1 Introduction

Glioblastoma multiforme (GBM) is the most common primary tumor of the CNS in adults and accounts for more than 50% of malignant gliomas [30]. Despite the advancement in therapies including surgical resection and improved chemo- and radiotherapy, the prognosis is poor with median survival period of 14.6 month [1,54,60]. Based on the gene expression profile and molecular signatures, GBM has been classified into four subtypes – classical, mesenchymal, proneural, and neural [22,44,58]. Understanding the biology of aggressive gliomas is very important to design better and effective strategies for treatment and to improve survival for GBM patients. However, one of the major hurdles in performing studies to identify targets and signaling pathways in aggressive tumors is limitation of experimental models. Much of the data accumulated over decades from preclinical studies have relied upon conventional 2D cell cultures using cancer cell lines. Though these studies have contributed towards enhancing our knowledge of gliomas, the model suffers from inherent disadvantage of not reflecting the structure and biology of *in vivo* tumors [7,42]. Multicellular spheroids (MCS) in contrast to 2D-monolayers are 3D structures and mimic many of features like the architecture, cell–cell interaction, oxygen and nutrient transport and conditions of *in vivo* tumors including the necrotic core [20,27]. Numerous studies have reported that spheroids display multi-drug resistance and are also resistant to radiotherapy compared to cells cultured as monolayers [15, 17]. MCS therefore serve as attractive model for a wide range of studies including as drug delivery, toxicity, and metabolism [31,34,39].

Prostate apoptosis response (Par)-4, a tumor suppressor was first identified in rat prostate cancer cells undergoing apoptosis in response to apoptotic stimuli [50]. Par-4 is a pro-apoptotic protein of approximately 38 kDa, encoded by PAWR gene (PKC apoptosis WT1 regulator) [38] and expressed ubiquitously in normal and cancer cells. Consistent with its tumor-suppressive activity, Par-4 is silenced or down regulated transcriptionally or post-transcriptionally in various types of cancers [14,40,45]. Several studies have documented the association of low level of Par-4 with poor prognosis in cancers of prostate [45,49,2] endometrial [40], renal [14], pancreas [2], and breast [41]. Par-4 has been shown to activate apoptosis through intrinsic and extrinsic pathways [4,10]. Upregulation or induction of Par-4 by apoptotic stimuli such as tumor necrosis factor alpha (TNFα), TRAIL [6] and Fas [11] induce cell death in cancer cells. Other studies showed that overexpression of Par-4 enhances the activity of anticancer drugs such as 5-fluorouracil [59,28] and induces radio-sensitivity [12]. While the intracellular role of Par-4 is established and the mechanisms well studied, recent studies have demonstrated that secretory or extracellular Par-4 induces apoptosis in cancer cells [9,46]. However, the potential of secretory Par-4 in drug-resistant tumors remains to be fully explored. We previously reported that upregulation of intracellular Par-4 and secretion of Par-4 were crucial for tamoxifen (TAM)-induced apoptosis in human glioma stem cells [25]. In the present study, we investigated the role of intracellular and secretory Par-4 in drug-induced apoptosis in human GBM cells using multicellular spheroids (MCS) as a model. We show that MCS derived from glioma cells are resistant to TAM-induced cytotoxicity and Par-4 secreted by TAM-treated glioma monolayers rendered MCS sensitive to TAM-induced cell death. Our findings also suggest the involvement of Akt and PKCζ in induction of secretory Par-4 and sensitization of MCS to TAM-mediated cytotoxicity.

## 2 Materials and methods

### 2.1 Ethics statement

The study was approved by the Ethics Committee of NCCS (Pune, India).

### 2.2 Chemicals

Tamoxifen, temozolomide, PKCζ pseudosubstrate inhibitory peptide and all fine chemicals were procured from Sigma–Aldrich (India) and PI3K inhibitor LY294002 was purchased from Calbiochem.

### 2.3 Cell culture

Human Glioma cell lines; LN-18 and LN-229 were maintained in Dulbecco’s modified eagle’s medium (DMEM) with 4 mM l-glutamine, 1.5 g/L sodium bicarbonate, 4.5 g/L glucose and supplemented with 5% heat-inactivated fetal calf serum (Gibco BRL, Carlsbad, CA, USA). HNGC-2 cells were cultured in DMEM medium supplemented with 5% fetal bovine serum (FBS,Gibco). Antibiotics (100 IU/ml penicillin and 100 μg/ml streptomycin (Sigma, USA) were added to the culture media. Cultures were maintained in 5% CO_2_ humidified incubator at 37 °C and cells grown for 24 h were used for experiments.

### 2.4 Development of primary cultures from tumor samples

GBM tumor samples were provided by D.Y. Patil Medical College and Inamdar Hospital (Pune). Grading of tumors was done according to WHO criteria by pathologist. Tumor tissues were finely chopped in DMEM/Ham’s F12 medium (1:1) containing antibiotics and enzymatically digested with zymefree and accutase (Sigma, Saint Louis, MO) for 2 h and passed through a cell strainer (100 μm; BD, Germany) to obtain single cell suspension. Cells were then washed and plated in DMEM/Ham’s F12 (1:1) medium with antibiotics and 10% FCS in petridishes. Primary cultures once established were maintained in complete DMEM medium supplemented with 10% FCS. In this study, primary cultures derived from GBM tumor-G1 was used and experiments were conducted with cells in passages between15 and 30.

### 2.5 Generation of multi-cellular spheroids (MCS)

The liquid overlay technique described earlier with slight modification was used to generate spheroids. Petridishes or plates were coated with 0.8% agarose in DMEM and allowed to solidify for 30 min at room temperature. Spheroids were generated by inoculating 6 × 10^5^ cells per 60 mM petridishes, or 10^4^ cells/well of 96 well plates in DMEM supplemented with 5% fetal calf serum. The spheroids were treated with drugs and processed for MTT assay or Western blotting.

### 2.6 MTT assay

The effect of tamoxifen and temozolomide on cell viability was assessed in glioma cell lines and primary cultures (G1) by MTT 3-(4,5-dimethyl-2-thiazolyl)-2, 5-diphenyl-2H-tetrazolium bromide assay. Cells were treated for 24 h with drugs or with DMSO (vehicle control). MTT (5 mg/ml) was added and formazan crystals formed were dissolved in 10% SDS along with 0.01 N HCl. The absorbance was measured at 570 nm with reference to 640 nm using microplate reader (Molecular Devices, SPECTRA max 250, USA) and percent viable cells was calculated considering values in control as 100%. Vehicle control cells showed cell viability >95%.

### 2.7 Annexin V and PI staining

Cells were treated with tamoxifen for 12 h and apoptosis was determined using Annexin-V apoptosis detection kit (BD Pharmingen, USA) according to the manufacturer’s protocol. Data was acquired with flow cytometry (FACS CantoII, Becton–Dickinson) and analysis was done using Cell Quest Pro software (Becton–Dickinson).

### 2.8 Mitochondrial membrane potential (MMP)

The mitochondrial membrane potential was measured using Mitocapture Apoptosis Detection Kit (Oncogene Research). Cells were treated and processed according to manufacturer’s protocol. Flowcytometric analysis for disruption of mitochondrial membrane potential was done using FL-1 parameter in BD–FACS Canto II. Increase in green fluorescence depicts breakdown in mitochondrial membrane potential.

### 2.9 Generation of reactive oxygen species (ROS)

Dihydroethidium (DHE) is a lipophilic cell-permeable dye that can undergo oxidation to ethidium bromide or a similar product in the presence of superoxide, and, to a lesser extent, hydrogen peroxide and hydroxyl radicals. DHE fluorescence was quantitated by flowcytometry analysis on FL2-channel.

### 2.10 Confocal laser scanning microscopy

Control and tamoxifen treated cells were fixed with 3.7% paraformaldehyde for 10 min and permeabilized with 0.2% triton X-100 for 5 min. Cells were washed with PBS three times at each interval. After blocking for 1 h in 3% BSA, cells were incubated with antibody to Par-4 (Santa Cruz Biotechnology, USA) for 2 h followed by goat anti-rabbit Cy3 labeled antibody or phalloidin for actin, (Molecular Probes, Invitrogen, USA) for 1 h at room temperature. DAPI was used to stain nucleus. Images were acquired using confocal laser scanning microscope (Carl Zeiss or Leica, Germany).

### 2.11 Western blotting

Control and treated cells were lysed using RIPA lysis buffer (120 mM NaCl, 1.0% Triton X-100, 20 mM Tris–HCl, pH 7.5, 100% glycerol, 2 mM EDTA and protease inhibitor cocktail, Roche, Germany). Total protein (30 μg) was electrophoresed on 10% SDS–polyacrylamide gels and blotted onto PVDF membrane (Millipore, Bedford, MA, USA). After blocking with 5% BSA at room temperature, the blots were probed with one of these antibodies: Par-4 (Cell signaling technologies, Santa Cruz Biotechnology, USA), PARP (Santa Cruz Biotechnology), GRP78 (Abcam, Cambridge, England), Akt, PKCζ, actin (Molecular probes, Invitrogen, USA), pAkt-ser 473, or pPKC ζ (Santa Cruz Biotechnology) overnight at 4 °C. The secondary antibodies were horseradish peroxidase-conjugated goat anti-rabbit IgG and goat-anti-mouse IgG (Bio-Rad, USA). The immunoreactive bands were visualized by chemiluminescence using Super Signal West Femto Maximum Sensitivity Substrate (Pierce, USA). GAPDH and actin were used to normalize levels of proteins detected.

For secretion of Par-4, HNGC-2, LN-18 and G1 cells were exposed to tamoxifen for 24 h and supernatants were collected and concentration (20-fold) was achieved using 30 kDa cut-off filters (Millipore, USA).

### 2.12 Real time PCR

RNA was isolated using TRIzol reagent (Invitrogen, USA) and reverse-transcribed into cDNA (Promega, Madison, WI) according to the manufacturer’s instructions. Quantitative real time PCR was performed using SYBR® Premix Ex Taq™ II (Takara, Japan) in Realplex Real-Time Thermal Cycler (Eppendorf). PCR reactions were performed in duplicates and program consisted of initial activation at 95 °C for 2 min followed by 40 cycles of denaturation at 95 °C for 15 s and primer annealing at 60 °C for 45 s. Melting curve analysis was used to determine the specific PCR products. All primers used for Real-Time PCR analysis were synthesized by Integrated DNA Technologies, India. GAPDH or 18s ribosomal RNA was used as an internal control. List of the primers is included in Table 1 of Supplementary data. The changes in the threshold cycle (C_T_) values were calculated by the equation: $-\Delta C_{T}=C_{T(\text{target})}-C_{T(\text{endogenous}\;\text{control})}$ and fold difference was calculated as $2^{-\Delta (\Delta C_{T})}$.

### 2.13 Transfections

LN-18 and LN-229 cells were cultured in 96 well, 6 well plates or on coverslips to 70% cell confluency. Cells were transfected with lipofectamine 2000 (Invitrogen Carlsbad, USA) and ON-TARGET plus SMART pool, human PAWR siRNA or non-targeting siRNA (Dharmacon, Inc., USA). After 48 h, the cells were treated with tamoxifen and assessed for cell viability by MTT assay or processed for analysis of Par-4 levels by Western blotting and immunofluorescence.

### 2.14 Immunohistochemical staining

The expression of PAR-4 was examined in brain glioblastoma tissue array (US Biomax, Inc). The samples included 40 samples in duplicates of GBM of different grades and normal brain tissue as control. Antigen retrieval was done by using citrate buffer, pH 6.0 by microwave method. The sections were cooled and washed with PBS before incubating in 3% aqueous hydrogen peroxide for 15 min to quench endogenous peroxidase activity. Nonspecific binding was blocked by incubation with serum for 30 min at room temperature. The sections were stained for Par-4 specific antibody (Sigma, USA) for 2 h at RT. The antigen–antibody complexes were detected by Dako kit (EnVision™ G|2 Doublestain System, Rabbit/Mouse (DAB+/Permanent Red Code K5361). Diaminobenzidine was used as a chromogen and sections were counterstained with hematoxylin. The sections were dehydrated, and mounted with a glass coverslip and DPX used as mounting media. Negative controls included sets processed similarly in the absence of Par-4 antibodies. The slides were viewed by expert pathologists and scored based on intensity of staining, negative staining (zero), low (+) medium (++) and high (+++).

### 2.15 RNA isolation and microarray analysis

LN-18 cells were cultured as monolayers or spheroids for 24 h and processed for RNA isolation. Samples in duplicates from two different passages were processed for microarray analysis. RNA was extracted using TRIzol reagent (Invitrogen, Carlsbad, USA) as per manufacturer’s instructions and RNA was quantified using Nanodrop VR spectrophotometer (NanoDrop Technologies, Wilmington, DE). Sample amplification was performed with 200 ng of total RNA using Agilent’s Quick Amp Labeling Kit One Color to generate complementary RNA (cRNA) for oligo microarrays. cRNA was processed for microarray analysis on a Whole Human Genome Oligonucleotide Microarray (G4112A, 41,000 genes; Agilent Technologies, Santa Clara, CA) according to the manufacturer’s instructions. Microarray experiments were performed at Genotypic Technology, (Bengaluru, India).

Microarray data analysis-Raw data was normalized and processed with GeneSpring.

GX10.0.2 software (Agilent Technologies). Genes with a fold change >1.5 and a *p*-value < 0.05 were considered as differentially expressed. Data analysis was done at Bionivid Technology, (Bengaluru, India). For data visualization, unsupervised hierarchical clustering was performed with the Pearson metric and average linkage method.

### 2.16 TCGA and REMBRANT data acquisition and processing

The Cancer Genome Atlas (TCGA) has the largest comprehensive data for different types of cancers. The TCGA project comprises multimodal data of glioma cases that includes Low grade gliomas (LGG), Glioblastoma Multiforme (GBM) classified into 4 subtypes- classical, mesenchymal, proneural and neural and normal samples. The data is available from the TCGA website (https://tcga-data.nci.nih.gov/tcga/). REMBRANDT is portal for data exclusively from brain tumors including GBM (228 cases) astrocytomas (148) oligodendrogliomas (67) and normal cases (21). The dataset was searched for GBM cases for level 3 gene expression based on the Agilent microarrays (Human gene U133A). The expression value of PAWR gene for each case was collected and cases were segregated as High (expression value ⩾−2), Low (expression value ⩽−2) and Intermediate value (expression value between ±1.2 and ±2.0) tumor samples. Survival period was calculated in days/months from the date of diagnosis to the time of death. From TCGA, we collected data for gene expression of LGG (27), GBM (97) and normal samples (10) and selected PAWR for analysis. The mean and SD values were calculated and analyzed for significance between groups and subtypes. Survival data of glioma cases from (Rembrandt data) was stratified into high, low and intermediate levels of expression of PAWR and KM plot for Highest intensity probe (204004_at) for samples were plotted. Comparison for survival probability between groups was done.

### 2.17 Statistical analysis

The data is represented as mean ± standard deviation. Independent *t*-test was used to calculate the difference of the data between two groups in viability assay. Chi-square test was used to evaluate the difference in the expression of Par-4 analyzed by immunohistochemistry. Kaplan–Meier estimates (log-rank test) were used to study if a variable was associated with the survival of glioma patients. *p*-values < 0.05 were considered significant.

## 3 Results

### 3.1 PAWR is differentially expressed in low and high grade gliomas

**Fig. 1:**
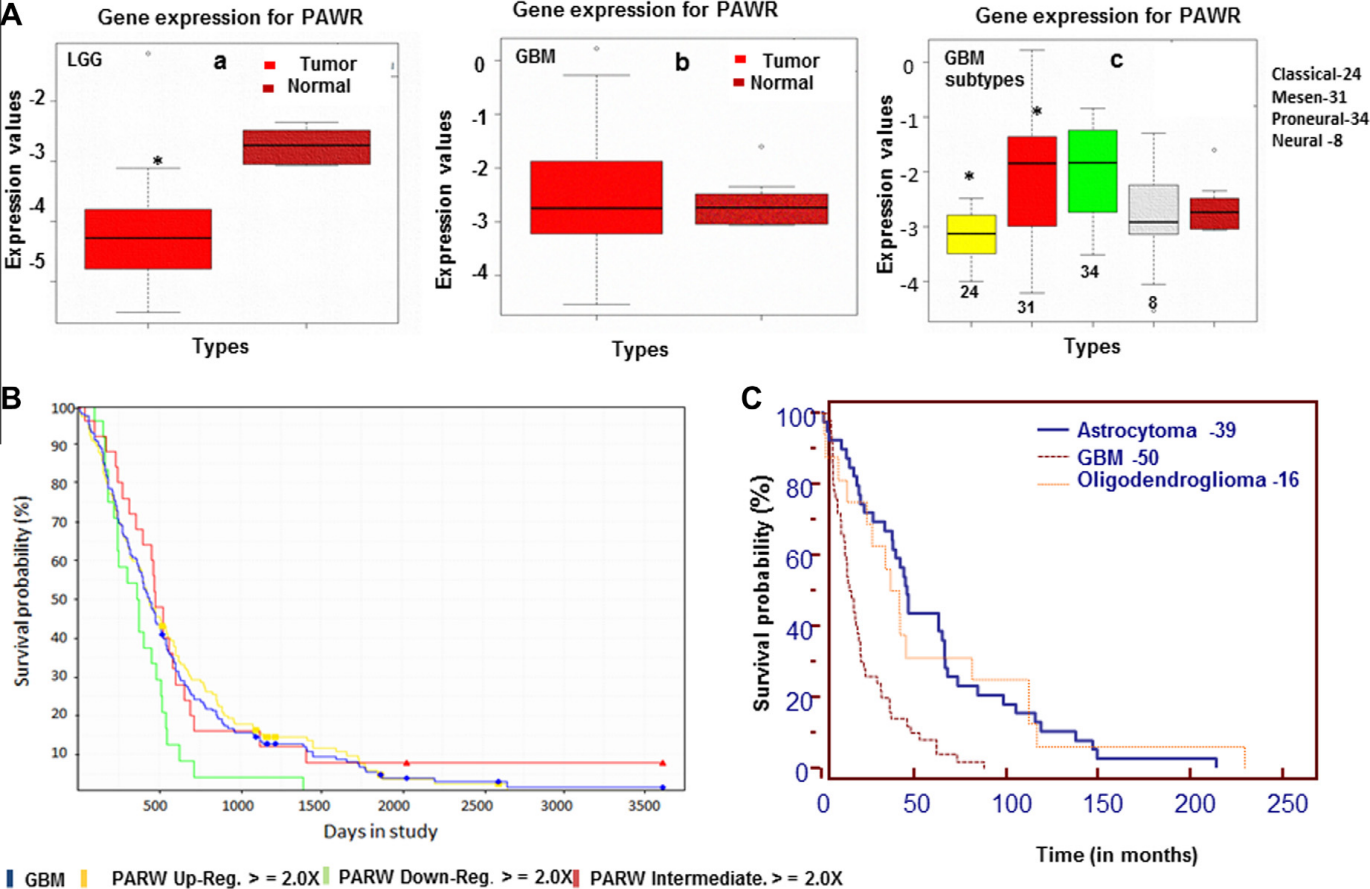
Expression of PAWR gene and survival analysis in gliomas (A) Box-plots depict comparison of PAWR expression between (a) Low grade gliomas (LGG) and normal brain tissue; (b) GBM and normal brain tissue; (c) subtypes of GBM and normal brain tissue. The data is derived from TCGA analysis on Agilent platform. Rembrandt data base comprising cases of GBM (228), astrocytoma (148) and oligodendroglioma (67) was downloaded and Kaplan–Meier estimates (log-rank test) were made (B) Survival graphs in GBM cases with low, intermediate and high level of PAWR gene (C) Comparison of survival probability in GBM, astrocytoma and oligodendroglioma cases with low expression of PAWR gene. Bars represent mean values ± SD, ^∗^*p* < 0.05 difference was between normal vs tumor groups.

The expression of PAWR was significantly lower (*p* < 0.05) in LGG compared to normal samples (Fig. 1A–a). GBM samples displayed a high degree of variation in PAWR expression and the level was not significantly different compared to normal group (Fig. 1A–b). Interestingly, analysis of GBM samples classified into subtypes [58] revealed that PAWR was upregulated (*p* < 0.036) in mesenchymal group and downregulated (*p* < 0.01) in classical with respect to normal, thus indicating a differential expression among GBM tumors (Fig. 1A–c). To examine the significance of Par-4 in survival, REMBRANDT data for gliomas was downloaded and samples were stratified for expression of PAWR into upregulated, downregulated and intermediate groups. Kaplan–Meier survival plots were drawn based on the expression levels of PAWR and analyzed by log-rank test. The survival period is significantly lower in cases with low level of PAWR (13.16 ± 9.00) compared to cases with upregulated (22.65 ± 24.82) and intermediate (20.17 ± 18,20) level (Fig. 1B). We further analyzed the data for association of PAWR and survival period in different types of gliomas. Samples with low expression (tumor samples with expression value ⩽ (mean (normal samples) − SD (normal samples) of PAWR were sorted within astrocytoma, oligodendroglioma and GBM groups. As depicted in Fig. 1C, low PAWR expression correlated with low survival period (21.85 ± 19.30) in GBM but not in astrocytomas (59.13 ± 47.26) and oligodendrogliomas (58.04 ± 59.80) suggesting that low PAWR is a predictive risk factor in GBM.

**Fig. 2:**
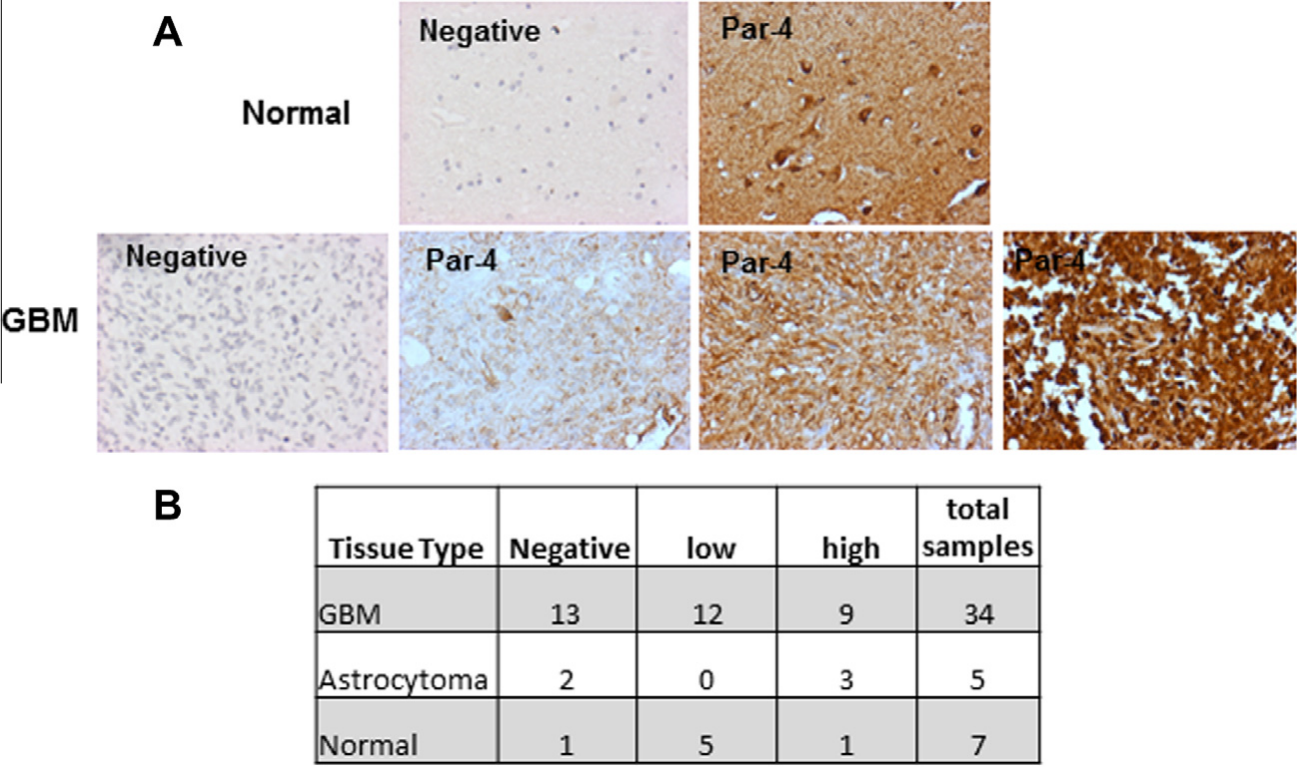
Expression of Par-4 in tissue samples of Gliomas and normal brain by immunohistochemical staining. (A) The panel shows negative control and one normal sample (above), negative control and three GBM samples showing different intensities of Par-4 expression (below). (B) The table summarizes the number and level of expression of Par-4 in GBM, normal and astrocytoma groups.

To analyze the expression of Par-4 protein in gliomas, immunohistochemical staining was done in tissue sections of array comprising grade III astrocytomas, GBM and normal brain samples. The samples were scored based on the intensity of staining. Normal brain tissues expressed detectable levels of Par-4, scored as low (+ or ++) in 5/7 samples (Fig. 2B). GBM showed variation with samples showing no staining (negative), moderate or high intensity of Par-4 (Fig. 2A). The expression levels of Par-4 was not significantly different between normal and GBM. The variation may be attributed to samples of different subtypes of GBM included in the array. These results are in line with the TCGA data in GBM.

### 3.2 Upregulation of genes associated with chemoresistance in MCS model of human GBM cells

**Fig. 3:**
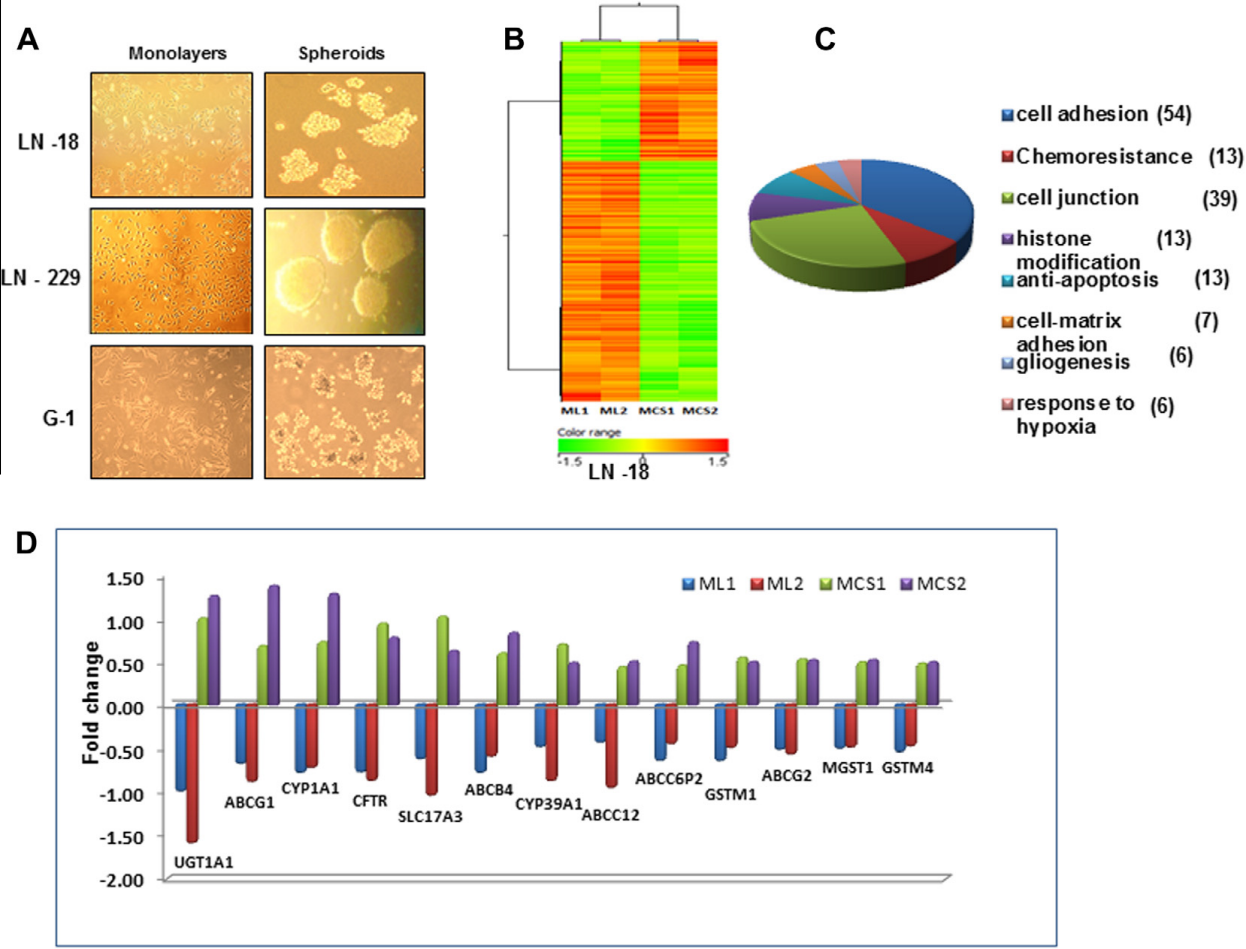
Differential gene expression patterns between monolayers (ML) and multicellular spheroids (MCS). (A) Human glioma cell lines-LN-18, LN-229 and G1 primary culture was grown as ML and MCS for 24 h. The images show the difference in size and morphology of MCS formed in the 3 cell types. (B) Hierarchal clustering profile of genes expressed (analyzed by microarray data) in ML and MCS derived from LN-18 cell line. The datasets represent two separate RNA samples for each of the culture models. (C) Graphical representation of selectively enriched gene ontology categories (*p* value ⩽ 0.05) in MCS analyzed with Gene spring software. The number of genes differentially regulated in the respective processes is mentioned in parentheses. (D) The graph depicts fold changes in expression of chemoresistance genes in ML and MCS of two data sets.

**Fig. 4:**
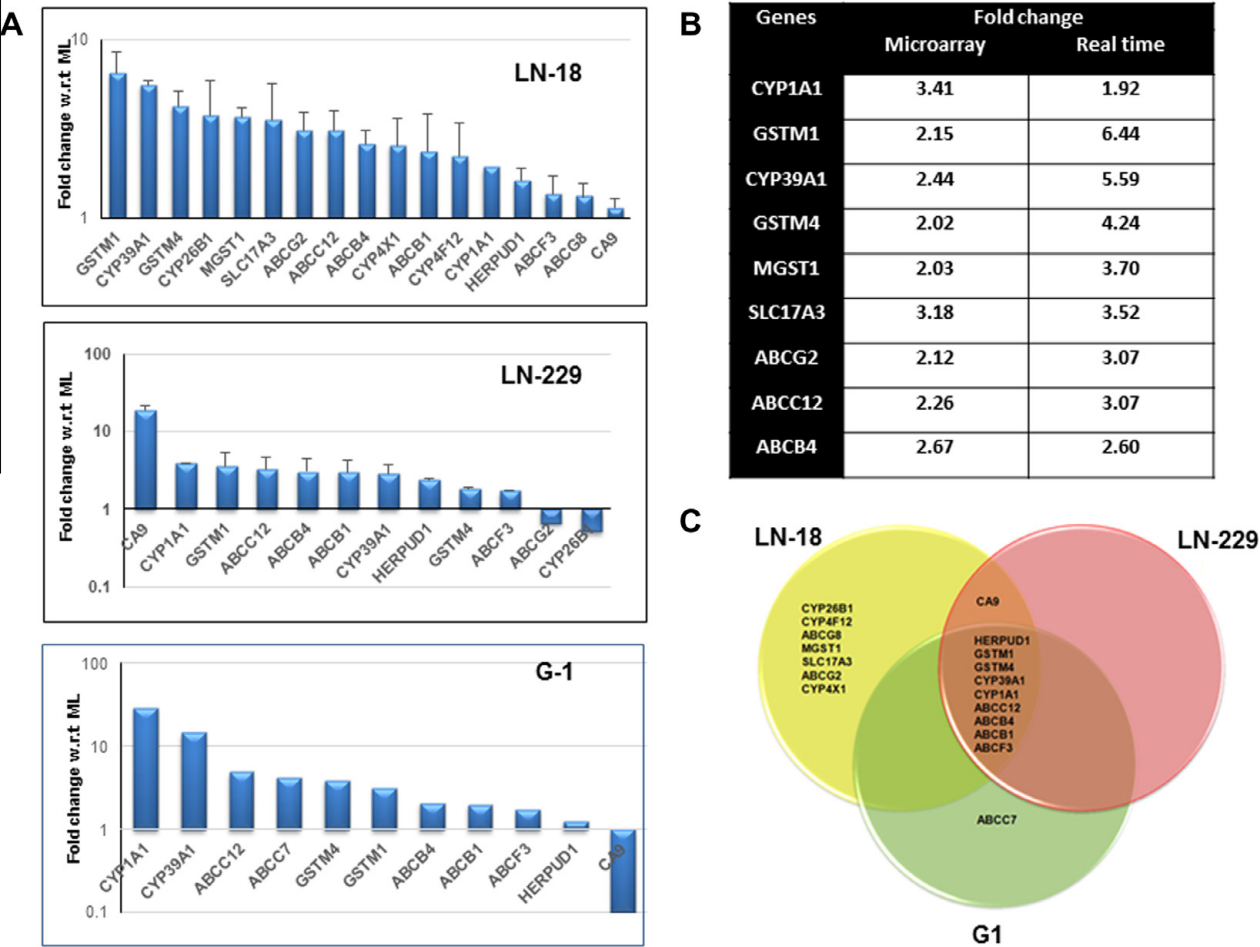
Regulation of genes related to chemoresistance genes in MCS (A) Transcript levels of chemoresistance genes in glioma cell lines and primary culture (G1) measured by quantitative real-time PCR (qRT-PCR). *Y*-axis represent fold change in MCS w.r.t ML. 18S rRNA or GAPDH was used as internal control. The data is mean ± of 3 independent experiments for LN-18 and LN-229 cells. (B) The table shows the comparison of fold changes in the expression of chemoresistance genes generated by microarray analysis and validated by qRT-PCR in LN-18 cell line. (C) The Venn diagram shows the genes upregulated in MCS among LN-18, LN-229 and G1 cultures.

Multicellular spheroids were generated from human glioma cell lines – LN-18, LN-229 and primary cultures-G1. It was interesting to note that even with equivalent cell number the spheroids were morphologically different. LN-229 cells formed very compact and large spheroids while LN-18 cells formed loose spheroids of different sizes. The primary culture-G1 formed small spheroids (Fig. 3A). Gene profiling analysis of LN-18 cells revealed differential expression of genes associated in MCS compared and cells in monolayers (Fig. 3B). Functional annotation of differentially expressed transcripts using Gene Ontology (GO) revealed that the number of genes most significantly altered (FC ⩾ 2 and *p* ⩽ 0.05) were in the categories of cell adhesion, cell junction and regulation of cell proliferation. Other groups included genes associated with cytoskeleton and actin organization (Fig. 3C). Cell–cell interactions resulting in cytoskeletal reorganization activate multiple signal transduction pathways that directly influence cell survival, growth and differentiation [21]. Multidrug resistant phenotype is reported to correlate with increased expression of genes associated with chemoresistance [57]. We analyzed the microarray data for alterations in the expression of chemoresistance genes in MCS. As shown in Fig. 3D genes associated with chemoresistance that were expressed at very low level in monolayers were upregulated in MCS. The functions of each of these chemoresistance genes are depicted in Supplementary data – Table S2. To validate the microarray data, transcript levels of the chemoresistance genes with inclusion of additional genes were measured in LN-18, LN-229 cell lines and G1 primary culture by quantitative real-time PCR (qRT-PCR). As shown in Fig. 4A, most of the chemoresistance genes analyzed were upregulated in MCS compared to cells grown as monolayer cells. The genes upregulated were similar in LN-229 and G1 cells while LN-18 cells showed upregulation of 7 additional genes. A comparison of the gene expression levels from microarray data and q-PCR is shown in table (Fig. 4B). Most importantly, nine genes that were overexpressed were common in the MCS of all three cell cultures. Fig. 4C indicating the relevance of upregulation of genes related to chemoresistance in MCS.

### 3.3 MCS are resistant to TAM-induced cell death

**Supplementary Figure 1:**
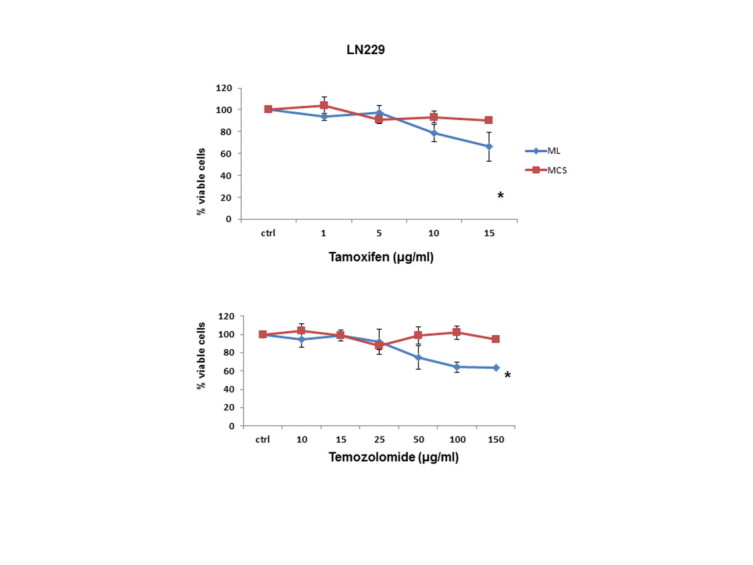
Effect of tamoxifen and temozolomide on ML and MCS . Graphs display dose dependent response of ML and MCS of LN-229 cells exposed to temozolomide and tamoxifen for 24 h. The effect was assessed by MTT assay. The cell viability of untreated control cells was considered as 100%. The data represents mean ± SE (*n* = 3). ^∗^*p* < 0.05 − difference between ML vs MCS.

**Fig. 5:**
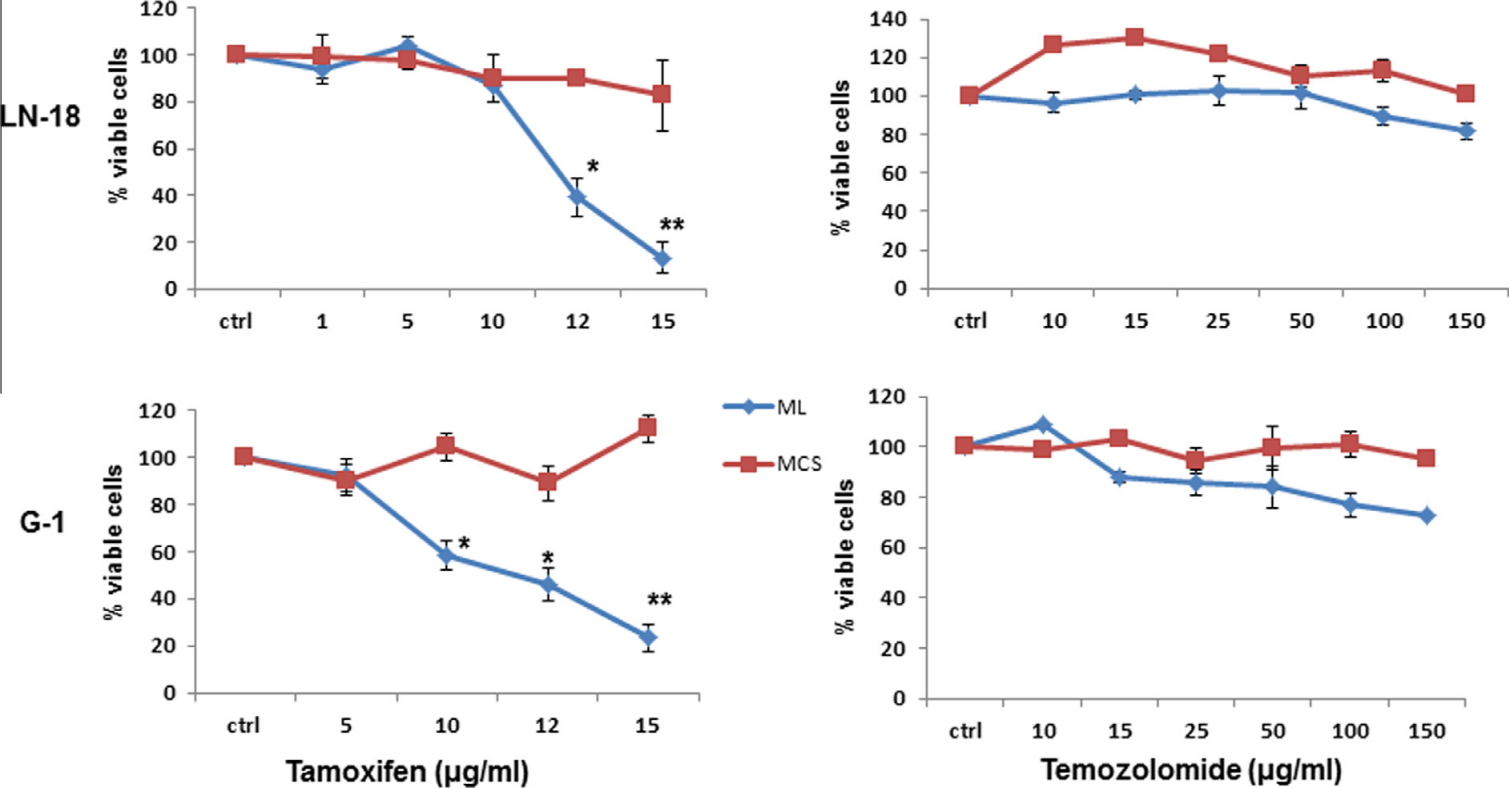
Dose dependent response of ML and MCS to tamoxifen and temozolomide. Graphs display dose dependent response of LN-18 and G1 grown as ML (blue) and MCS (red) to tamoxifen and temozolomide. The effect was assessed by MTT assay. Cell viability of untreated control cells was considered as 100%. The data represents the mean ± SE (*n* = 3). ^∗^*p* < 0.01 difference between ML vs MCS. (For interpretation of the references to colour in this figure legend, the reader is referred to the web version of this article.)

To assess the response of monolayers and MCS to TAM and TMZ, we performed MTT viability assay in LN-18, LN-229 and G1 cells. Dose response curves showed that the cell lines were more sensitive to TAM than TMZ. We also observed that MCS of the cell lines and G1 were resistant even at 15 μg/ml of TAM (Fig. 5 and Supplementary Fig. 1). These preliminary data suggested that MCS from glioma cell lines and from primary cultures of GBM tumor are resistant to TAM-induced cytotoxicity.

### 3.4 MCS display low level of Par-4 compared to cells in monolayer

**Fig. 6:**
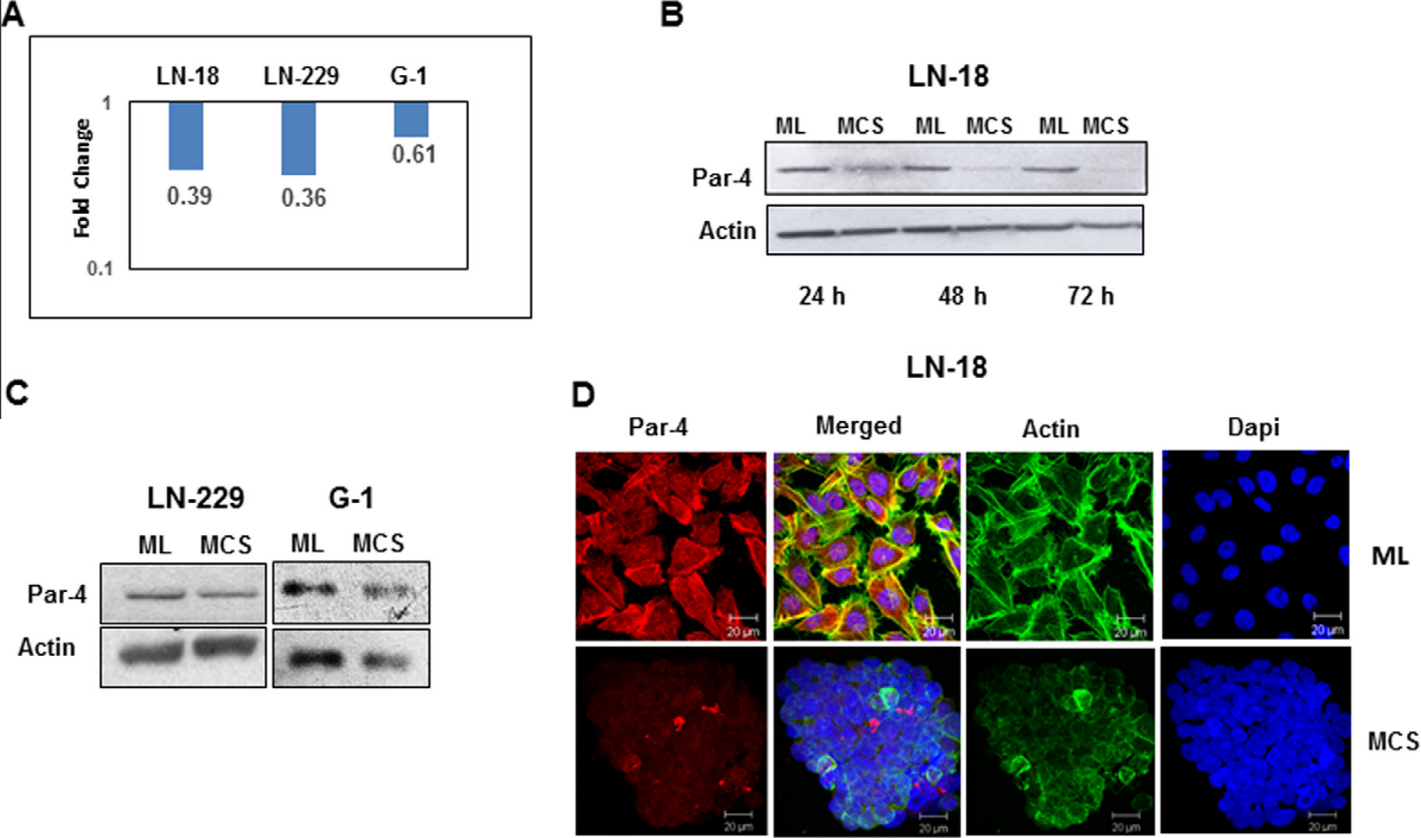
Gene and protein expression of Par-4 in ML and MCS. (A) The graph represents fold changes (logarithmic scale) of Par-4 gene quantified by qRT-PCR. Y-axis represents fold change in MCS w.r.t ML (B) Protein levels of Par-4 measured by Western blotting in LN-18 cells cultured as ML and MCS for different time periods (C) Protein expression of Par-4 in LN-229 and G1 cultures at 24 h by Western blot analysis. Actin was used as loading control. (D) Immunofluorescence staining for Par-4 (red), actin (green) and nucleus (blue) of LN-18 cells grown as ML (above) and MCS (below). (For interpretation of the references to colour in this figure legend, the reader is referred to the web version of this article.)

Par-4 is a pro-apoptotic protein and is upregulated in response to many apoptosis stimuli [47,53]. Based on our preliminary observations that MCS display high levels of chemoresistance genes and are resistant to TAM-induced cell death, it was of interest to examine the expression of Par-4 in the two culture models. As depicted in Fig. 6A, MCS of LN-18, LN-229 and G1 cultures showed a remarkable reduction in Par-4 transcript compared to monolayer and the effect was more evident in G1 cells. Whole cell lysates of LN-18 cells cultured as MCS showed down regulation of Par-4 and was not significantly different in MCS cultured for 24, 48 and 72 h (Fig. 6B). Further results with Western blotting and immunofluorescence staining in LN-229, G1 and LN-18 cells confirmed reduced Par-4 expression in MCS compared to cells grown as monolayers (Fig. 6C and D).

### 3.5 TAM upregulates Par-4 expression and induces cell death in cells grown as monolayers but not in MCS

**Supplementary Figure 3:**
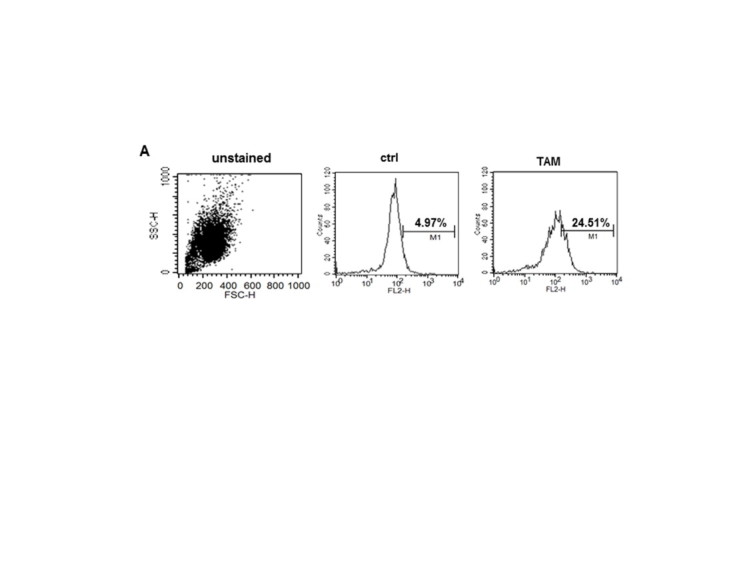
Reactive Oxygen Species (ROS) generation and TAM-induced apoptosis in LN-18 cells (A) LN-18 cells were treated with TAM for 24 h and analyzed for ROS generation with Dihydroethidium (DHE) probe followed by flow cytometry analysis. Figure is representative of two similar experiments.

**Fig. 7:**
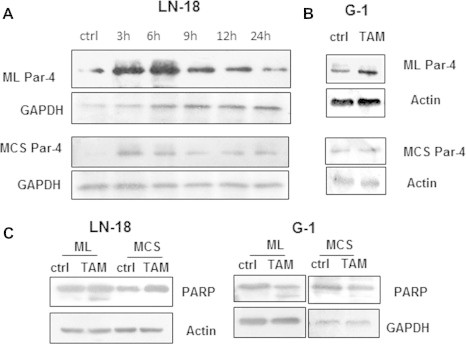
MCS are resistant to TAM-induced apoptosis. (A) LN-18 cells were treated with TAM for different time periods and analyzed for Par-4 protein levels in ML (above) and MCS (below). (B) Protein expression of Par-4 in G1 cells cultured as ML (above) and MCS (below). (C) ML and MCS derived from LN-18 and G1 were treated with TAM and cell lysates were probed for PARP cleavage.

Further experiments were performed to examine the effect of TAM on the expression of Par-4 in the two culture models. Par-4 level increased at 3 and 6 h in monolayers and MCS and gradually decreased with time, however the increase was more robust in monolayers (Fig. 7A). In G1 primary cells, increased expression of Par-4 was observed with TAM at 24 h in monolayers but there was no change in MCS (Fig. 7B) indicating a difference in kinetics in Par-4 expression in the two cultures systems. To assess the effect on apoptosis, the cell lysates were monitored for PARP cleavage. As shown in Fig. 7C, cleaved PARP was detected in LN-18 and G1 cells grown as monolayers but not in MCS. To examine the involvement of Par-4 in apoptosis, LN-18 and LN-229 cells transfected with Par-4 specific siRNA and control siRNA were exposed to TAM for 24 h and cell viability was measured by MTT assay. As depicted in Supplementary figures – Figs. 2 and 3, TAM induced apoptosis as quantitated by Annexin-V/PI staining and breakdown in mitochondrial membrane potential in these cell lines. Also TAM (10 μg) increased the necrotic population in LN-18 cells. Staining of cells with Dihydroethidium (DHE) revealed that 24.51% cells were positive for ROS compared to control cells (4.97%, Supplementary Fig. 3) suggesting that generation of ROS might have contributed to necrosis in cells exposed to TAM. Par-4 silenced cells showed higher cell viability in TAM-treated cells compared with control-non-target siRNA suggesting the role of Par-4 in TAM-induced apoptosis.

### 3.6 TAM induces secretory Par-4 and downregulates levels of Akt and PKCζ in monolayers but not in MCS

**Fig. 8:**
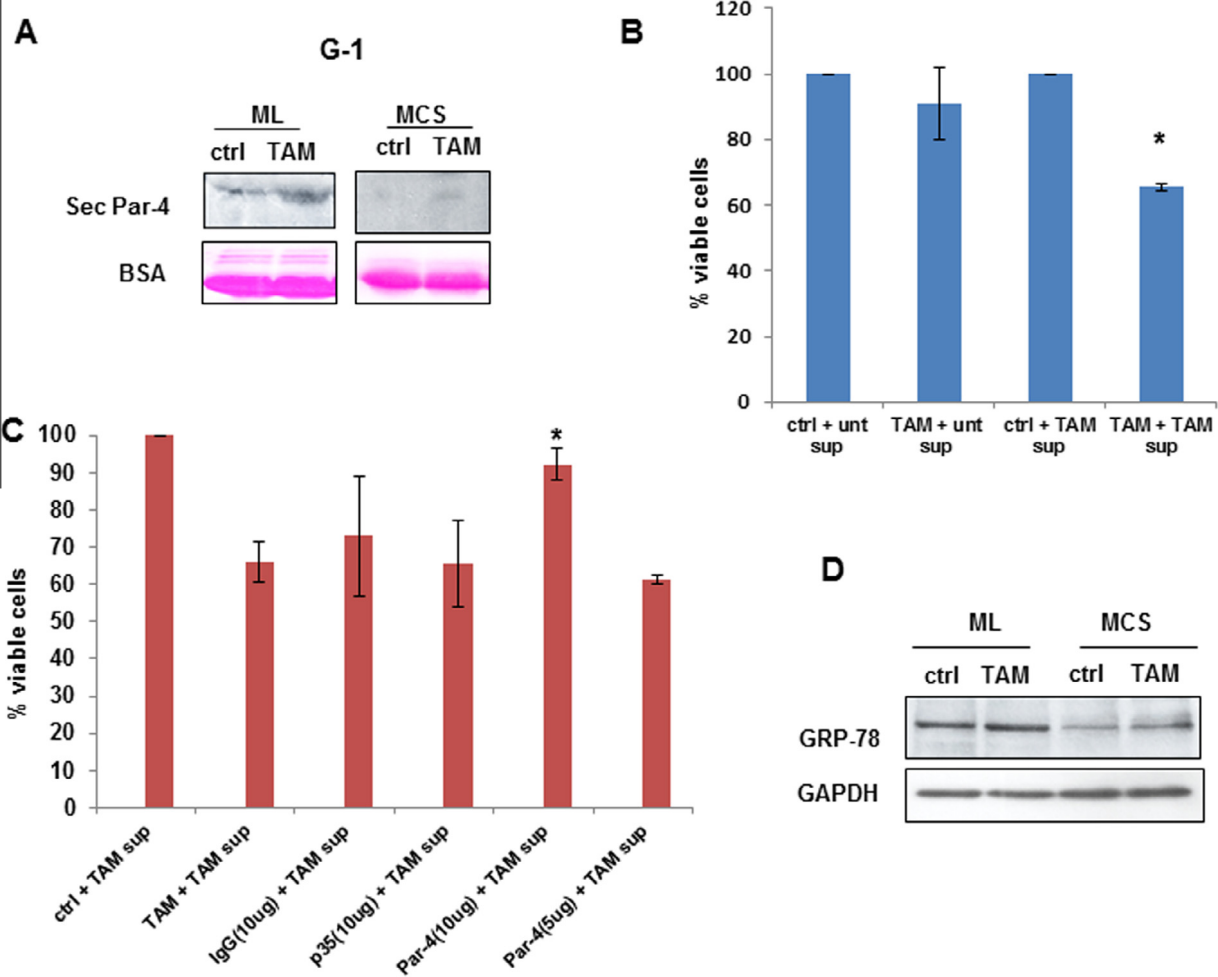
TAM induces secretory Par-4 in ML but not MCS. (A) G1 cells cultured as ML and MCS were treated with TAM for 24 h and supernatants were analyzed for secretory Par-4 by Western blotting. The blots were stained with Ponceau and BSA was used as loading control (lower panel). (B) MCS of G1 were treated with combination of TAM and secretory Par-4 derived from HNGC-2 cells exposed to TAM (described in Section 2). Supernatant of untreated HNGC-2 cells was used as control. Cell viability was assessed after 24 h by MTT assay. (C) MCS of G1 cells were exposed to supernatant containing Par-4 that was pre incubated for 30 min with Par-4 antibody and cell viability was assessed by MTT assay. Antibody to p35 and species specific IgG were used as internal control. (D) Expression of GRP78 in ML and MCS of TAM treated G1 cells was assessed by Western blotting. GAPDH was used as loading control. ^∗^*p* < 0.05 is difference in viability between cells-treated with TAM in combination with conditioned medium containing Par-4 vs control supernatant (Fig. B) and in the presence of Par-4 antibody vs control antibody(Fig. C).

We have previously demonstrated the role of secretory Par-4 in TAM-induced apoptosis in glioma stem cell line-HNGC-2 [25]. To address the question to what might be the factors that contribute to sensitivity/resistance in monolayers and MCS, we chose to analyze the conditioned medium (CM) of G1 cells treated with TAM. Interestingly, significant level of Par-4 was detected in supernatants of cells cultured as monolayer but not in MCS (Fig. 8A). Based on these data, we speculated whether addition of exogenous Par-4 would render MCS sensitive to TAM-induced apoptosis. For this purpose, we treated MCS of G1 cells with TAM in the presence of CM derived from HNGC-2 cells treated with TAM and assessed for cell viability. We observed that MCS exposed to TAM showed reduced viability in the presence of conditioned medium (CM) containing secretory Par-4 but not control CM. Also, treatment with CM-containing secretory Par-4, alone did not affect cell viability (Fig. 8B).

**Supplementary Figure 4:**
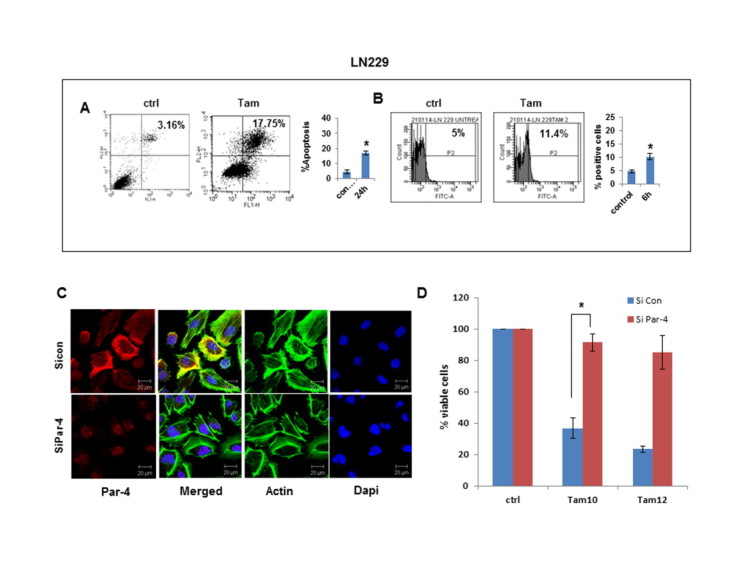
TAM-induced apoptosis and role of Par-4 in LN-229 cells (A) LN-229 cells were treated with TAM for 24 h and analyzed for apoptosis by staining cells with Annexin V and PI followed by flow cytometry analysis. (B) Cells exposed to TAM for 6 h were stained using Mitotracker kit and Mitochondrial membrane potential (MMP) was measured by flowcytometry. (C) LN-229 cells were transfected with control siRNA or Par-4 siRNA (100 nm) and analyzed by immunofluorescence (D) Cells transfected with Par-4 specific siRNA and control siRNA were exposed to TAM for 24 h and cell viability was assessed by MTT assay. The viability of siRNA control cells was considered as 100%. Data represent mean ± SE of three independent experiments.^∗^*p* < 0.05- treated vs. untreated control for figures a and b and Par-4 specific siRNA vs control siRNA in figure d.

To further confirm the involvement of secretory Par-4, CM of HNGC-2 cells exposed to TAM was incubated with antibody to Par-4 and the mixture was added to G1 cells in combination with TAM. As shown in Fig. 8C, a significant recovery was observed with Par-4 antibody that was concentration dependent but not with irrelevant (p35) or control antibody. Similar results were obtained in LN-18 cells (Supplementary Fig. 4). These findings suggest that secretory Par-4 and TAM-mediated intracellular signaling are both essential for inducing apoptosis. Extracellular Par-4 is reported to induce apoptosis by binding to the stress response protein, glucose-regulated protein-78 (GRP78). We found that MCS of G1 cells express lower level of GRP78 compared to monolayers and TAM did not affect the expression of GRP78 in both the models (Fig. 8D).

**Fig. 9:**
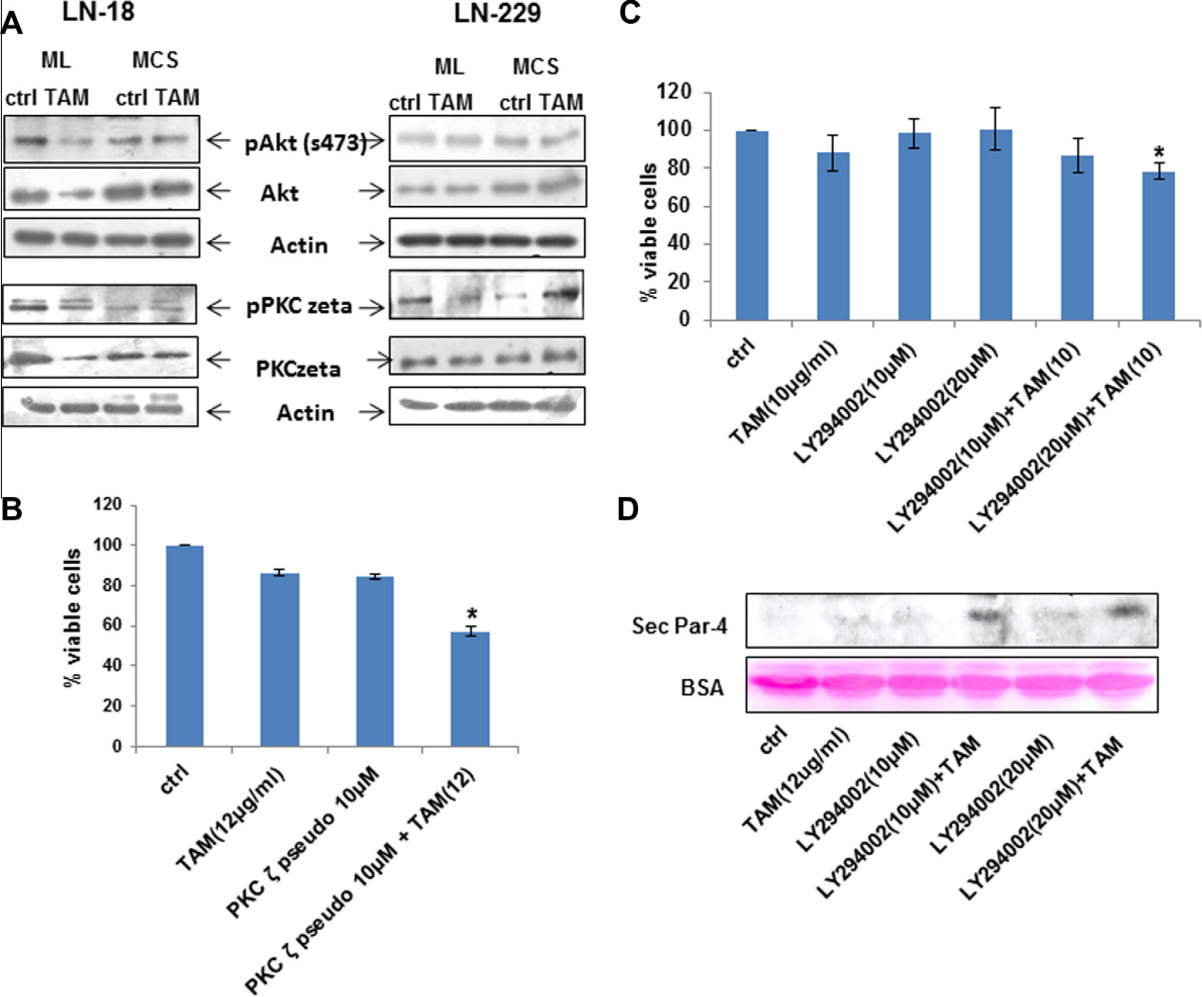
Role of Akt and PKCζ in ML and MCS (A) LN-18 and LN-229 cells cultured as ML and MCS were treated with TAM for 24 h and cell lysates were analyzed for expression of phosphorylated and total Akt and PKCζ. Actin was used as loading control. (B) LN-18 MCS were treated with combination of TAM (10 μg/ml) and PKCζ pseudosubstrate inhibitor for 24 h and assessed for cell death. The data represents mean of two independent experiments. (C) Also LN-18 MCS were treated with combination of TAM (10 μg/ml) and PI3K inhibitor (LY 294002) for 24 h and assessed for cell death. The data represents mean of two independent experiments. (D) Western blot represents the band of secretory Par-4 when MCS of LN-18 treated with combination of TAM (12 μg/ml) and PI3K inhibitor (LY 294002) for 24 h. Lower panel shows BSA as loading control. ^∗^*p* < 0.05 is difference in viability between cells-treated with TAM in combination with PKCζ pseudosubstrate inhibitor/LY 294002.

PKCζ is a binding partner of Par-4 and affects its proapoptotic function via Akt regulation [26]. Treatment of TAM in LN-18 and LN-229 cells resulted in reduced levels of PKCζ and Akt in lysates of monolayers but not in MCS (Fig. 9A). To test whether Akt contributed to resistance in MCS, we assessed cell viability in response to TAM in combination with inhibitors to PI3K (LY294002) and PKCζ (PKCζ pseudosubstrate inhibitory peptide). As shown in Fig. 9B, TAM-induced cell death was significantly enhanced in the presence of both these inhibitors. Collectively, these results suggest the involvement of secretory Par-4 as well as intracellular signaling involving Akt and PKCζ in rendering MCS sensitive to TAM-induced apoptosis.

## 4 Discussion

A correlation between high levels of Par-4 and better survival period has been reported in pancreatic cancers [2] and breast cancer [41]. Our data using the TCGA and REMBRANT data portals reveals association of high PAWR expression with survival in gliomas and suggests low PAWR level as a predictive risk factor for GBM but not oligodendroglioma and astrocytoma groups. A correlation between Par-4 expression and longer median survival is reported in high-grade gliomas that are IDH1 wild type [35].

One of the factors associated with failure of preclinical studies with anti-cancer agents has been the limitations in appropriate experimental models. In this study, we used MCS generated from human cell lines and primary cultures of GBM tumor to study the role of Par-4 in drug resistance. Interestingly, though the MCS differed in compactness and size in the cell lines and primary cultures, 9 genes including ABC transporter family members and Glutathione S-transferases (GSTs) that are involved in multi-drug resistance [48] were common in the three cultures. Importantly, MCS from the GBM cell lines and primary expressed low level of Par-4 transcript and protein suggesting an inverse correlation with chemoresistance genes. These data support the suitability of MCS as a model to study the role of Par-4 in drug resistance.

Temozolomide, an alkylating agent is the front line drug for treatment of GBM. It has been approved in the European Union for the treatment of patients showing progression or recurrence after standard therapy [18]. However, only 11% of the patients remain progression free at 2 years of treatment with standard therapy incorporating temozolomide [55]. Consistent with these studies, we found monolayers as well as MCS of GBM cell lines and primary cultures of GBM resistant to high doses of TMZ. Recent studies suggest that high doses of tamoxifen can be beneficial in the treatment of gliomas [43,52]. TAM is being evaluated in clinical trials for treatment of patients with malignant gliomas [24]. In our *in vitro* culture models, in contrast to monolayers that were sensitive, MCS were resistant to TAM-induced cell death, reaffirming chemoresistance in MCS. Recent studies demonstrated that TAM could significantly reduce the MDR in a variety of human cancers [36]. Par-4 level is enhanced in response to apoptotic stimuli by anticancer agents in wide variety of cancer cells [59,53]. TAM enhanced the expression of Par-4 in both cultures systems, though more robustly in monolayer cells, apoptosis was induced in monolayers but not in MCS suggesting that upregulation of Par-4 is not sufficient for inducing cell death. Recent studies have reported the role of secretory Par-4 in apoptosis triggered by stimuli causing endoplasmic reticulum stress in mammalian cells [10]. Our results that TAM effectively enhanced the expression of intracellular but not secretory Par-4 in MCS led us to hypothesize that secretory Par-4 is essential for inducing cell death in MCS. In this context, we observed that MCS was rendered sensitive to TAM-induced apoptosis in the presence of conditioned medium that contained Par-4 derived from HNGC-2 cells exposed to TAM. Furthermore, the effect was abrogated on pretreatment of conditioned medium with Par-4 specific antibody confirming that the involvement of secretory Par-4 in apoptosis stimulated by TAM. Collectively, these findings suggested that extrinsic Par-4 is effective in enhancing sensitivity of drug-resistant MCS to TAM-induced apoptosis.

The mechanism of induction of apoptosis by extracellular Par-4 involves interaction with cell surface GRP78 [51]. GRP78 is overexpressed in a variety of tumors and confers resistance to cytotoxic therapy [62,23]. It is generally present as an endoplasmic reticulum protein but its expression as a surface protein specifically in tumor but not normal cells, makes it attractive as potential target for anti-cancer therapy [63,29,33]. Previously, we reported that extrinsic Par-4 induces apoptosis in human glioma stem cell line HNGC-2 and the mechanism involved GRP78 [25]. In contrast to these observations, we found that in MCS, Par-4 containing supernatant alone could not induce apoptosis. We speculate that Par-4 was ineffective due to low level of GRP 78 in MCS. Though we have no direct evidence it is possible that low Par-4 expression led to decreased GRP78 level as reported in trophoblastic cells [13]. The expression of GRP78 is enhanced in response to a variety of ER stress inducers such as glucose starvation or hypoxia [19,33]. Tamoxifen induce endoplasmic reticulum stress [3] and enhance cytotoxicity of anti-cancer drug nelfinavir in breast cancer cells [8]. On these lines, it is reasonable to infer that the combination of TAM and secretory Par-4 is effective in inducing cytotoxicity in MCS by different mechanisms. While TAM does not significantly enhance GRP78 in MCS, it induces endoplasmic reticulum stress (as evidenced by caspase-12 activity-data not shown) and secretory Par-4 interacts with surface GRP78 complementing the action of TAM.

Further studies were directed towards identifying the possible factors/molecules that may be crucial in enhancing TAM-induced cytotoxicity. Activation of Akt and ERK42/44 signaling pathways are important in drug resistance [5,37]. In pancreatic tumors, Par-4 is known to act as a negative regulator of Akt activation via PKC zeta [56,26]. PKCζ is highly expressed in gliomas [61] and is associated with Par-4 [16]. It is noteworthy that TAM reduced the expression of Akt and PKCζ in GBM cells cultured as monolayer but not in MCS. Furthermore, inhibitors to PI3K/Akt or PKCζ enhanced TAM-induced cell death in MCS suggesting the involvement of Akt-mediated signaling in the process. Another study reported sensitization of glioma cells to tamoxifen-induced apoptosis by Pl3-kinase inhibitor mediated via the GSK-3β/β-catenin signaling pathway [32].

In conclusion, the present study has shown that secretory Par-4 sensitizes resistant glioma cells to TAM-induced apoptosis by mechanism involving Akt and PKCζ. Considering that little success has been achieved with inhibitors targeting PI3K/Akt for cancer therapy and TAM being evaluated in clinical trials for treatment of malignant gliomas, our findings suggest that secretory Par-4 can be induced by a combination treatment of TAM and Akt inhibitors to effectively kill cancer cells. However, further studies are warranted to identify the precise mechanism involved in secretion of Par-4 mediated by PI3K/Akt pathways. Since secretory Par-4 functions by binding to membrane GRP78, which is overexpressed in most cancer cells but not normal cells, secretory Par-4 is an attractive candidate for potentially overcoming therapy-resistance not only in malignant gliomas but in broad spectrum of cancers.

## Conflict of interest

None declared.

## Author contributions

Conceived and designed the experiments: PS, JCJ, PD and RS. Performed the experiments: JCJ, PD, RS, GC, KN and AM. Analyzed the data: PS, JCJ, PD, RS, AD, DB, GC, KN, BK and SK. Contributed reagents/materials/analysis tools: AC DR. Wrote the manuscript: PS, JCJ, PD and RS. Other (please specify): none.

## Figures and Tables

**Supplementary Figure 2:**
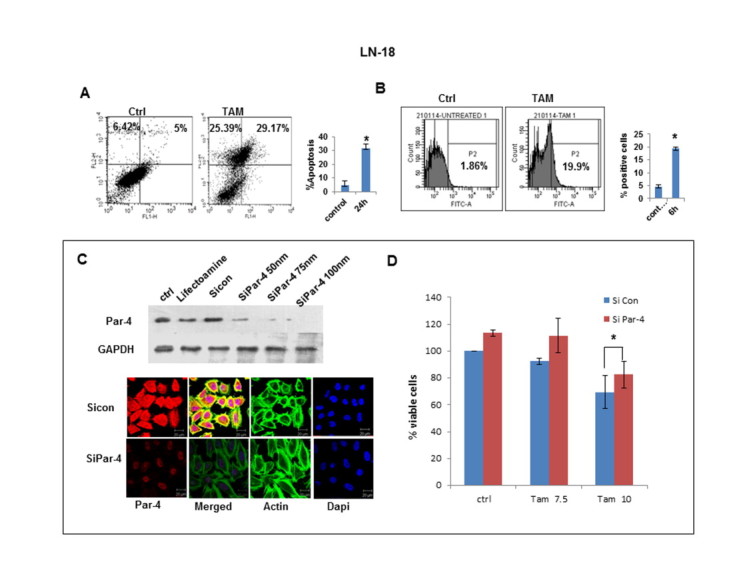
TAM-induced apoptosis and role of Par-4 in LN-18 cells (A) LN-18 cells were treated with TAM for 24 h and analyzed for apoptosis by staining cells with Annexin V and PI followed by flow cytometry analysis. (B) Cells exposed to TAM for 6 h were stained using Mitotracker kit and Mitochondrial membrane potential (MMP) was measured by flowcytometry. (C) LN-18 cells were transfected with control siRNA or serial concentrations of Par-4 siRNA and analyzed for Par-4 expression by immunoblotting, 100 nm Par-4 siRNA was used for immunofluorescence experiments. (D) Cells transfected with Par-4 specific siRNA and control siRNA were exposed to TAM for 24 h and cell viability was assessed by MTT assay. The viability of siRNA control cells was considered as 100%. data represent mean ± SE of three independent experiments.^∗^*p* < 0.05- treated vs. untreated control for figures A and B and Par-4 specific siRNA vs. control siRNA in figure D.

**Supplementary Figure 5:**
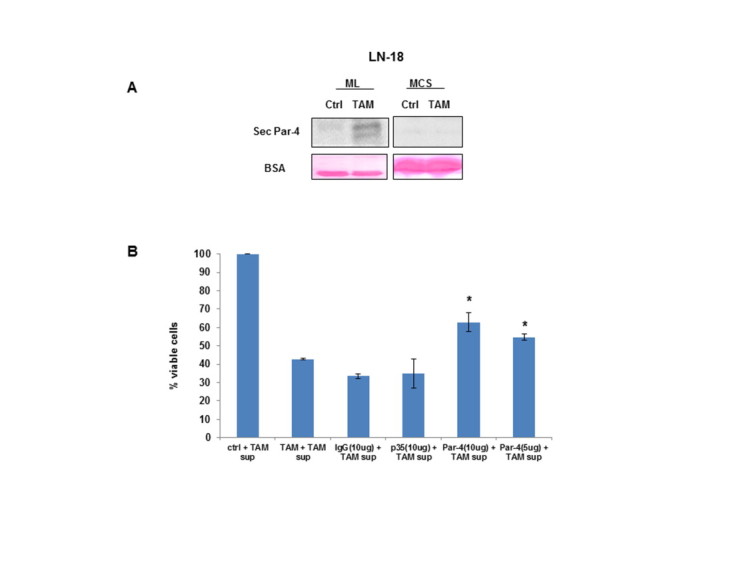
Secretory Par-4 renders MCS sensitive to TAM-induced apoptosis.(A) LN-18 cells grown as ML and MCS were treated with TAM and supernatants were assessed for secretory Par-4. The lower blots show Ponceau staining of BSA, used as loading control. (B) MCS of Ln-18 cells were exposed to supernatant containing Par-4 that was pre-incubated for 30 min with Par-4 antibody and cell viability was assessed by MTT assay. Antibody to p35 and species specific IgG were used as internal control. *p* < 0.05 in the presence of Par-4 antibody vs control antibody.
