# Supplementary material for: Secretory prostate apoptosis response (Par)-4 sensitizes multicellular spheroids (MCS) of glioblastoma multiforme cells to tamoxifen-induced cell death
Source: FEBS Open Bio. 2014 Nov 21;5:8–19. doi: 10.1016/j.fob.2014.11.005 (PMC4309838; doi:10.1016/j.fob.2014.11.005)
Supplement: Supplementary Tables 1 and 2 [file mmc1.docx]

**Supplementary Data – Tables 1 and 2**

Table-1**.** List of primers for detection of chemoresistant genes.

The sequence of the primers used:

| GSTM1 | Forward :5’ CCCAAGACCTGTGTTCTCAAA 3’  Reverse: 5’ GTAATGATGGGAGGGGATGTT 3’ |
| --- | --- |
| CYP39A1 | Forward : 5’ TAGCCCACAGAAAAGAAGCAA 3’  Reverse: 5’ TCCGCTGAAGGAGTAAGAACA 3’ |
| GSTM4 | Forward : 5’ GTCTGCAGAATCGACACCAAC 3’  Reverse: 5’ CGTCCCCCATCGTATACTTCT 3’ |
| CYP26B1 | Forward : 5’ ACACGGTGTCCAATTCCATT 3’  Reverse: 5’ GCCTCCTGGTACACGTTGAT 3’ |
| MGST1 | Forward : 5’ GCAAAGGAGAAAATGCCAAG 3’  Reverse: 5’ TACAGGAGGCCAATTCCAAG 3’ |
| SLC17A3 | Forward : 5’ TCTGCTGCCTTCTCTGGTTT 3’  Reverse: 5’ ACCCGACCTGTTGTTTCAAG 3’ |
| ABCG2 | Forward : 5’ GTGGCCTTGGCTTGTATGAT 3’  Reverse: 5’ GATGGCAAGGGAACAGAAAA 3’ |
| ABCC12 | Forward : 5’ CCAAGACTGACACCCTGGTT 3’  Reverse: 5’ GCAAGGACTTCAGGCTTGTC 3’ |
| ABCB4 | Forward : 5’ AGAATGGCCCTACTTTGTCGT 3’  Reverse: 5’ CTGGTCCAAAAATCGCTATGA 3’ |
| CYP4X1 | Forward : 5’ GGAAAAGGACTAGCGGCTCT 3’  Reverse: 5’ CAGAATGAGCCATCACCTCA 3’ |
| ABCB1 | Forward : 5’ CAGAGGGGATGGTCAGTGTT 3’  Reverse: 5’ CGTGGTGGCAAACAATACAG 3’ |
| CYP4F12 | Forward : 5’ TCCTACAGAGGAGGGCTTGA 3’  Reverse: 5’ TGATAGACCGGATGGTGTCA 3’ |
| CYP1A1 | Forward : 5’ CTTCCGACACTCTTCCTTCG 3’  Reverse: 5’ ATAGCACCATCAGGGGTGAG 3’ |
| HERPUD1 | Forward : 5’ CCCTCCTTTATGAGCACAGC 3’  Reverse: 5’ CCTCCAACAGCTACAGCACA 3’ |
| ABCF3 | Forward : 5’ CTCAACCAGCAGCGTGAATA 3’  Reverse: 5’ CTTCAGCTCAGGCAGCTTCT 3’ |
| ABCG8 | Forward : 5’ ACGCCATCTACCTCATCGTC 3’  Reverse: 5’ GCGTGAATCACCAGTCTTGA 3’ |
| CA9 | Forward : 5’ GTCTCGCTTGGAAGAAATCG 3’  Reverse: 5’ TTGGAAGTAGCGGCTGAAGT 3’ |
| PAR-4 | Forward : 5’ GCAGATCGAGAAGAGGAAGC 3’  Reverse: 5’ GCAGATAGGAACTGCCTGGA 3’ |
| GAPDH | Forward : 5’ ATGGGTGGAATCATATTGGAA 3’  Reverse: 5’ GAAGGTCGGAGTCAACGGATTT 3’ |
| 18S | Forward : 5’ AAACGGCTACCACATCCAAG 3’  Reverse: 5’ CCTCCAATGGATCCTCGTTA 3’ |

Table-2. Functional annotation of chemoresistance genes upregulated in MCS

| **Gene** | **Full name** | **Function** |
| --- | --- | --- |
| ***GSTM1*** | Glutathione S-transferase mu 1 | GSTs can operate in synergy with efflux transporters to confer resistance to several anticancer drugs. |
| ***GSTM4*** | Glutathione S-transferase mu 4 |  |
| ***ABCB1*** | ATP-binding cassette, sub-family (MDR), member 1 | ABC transporter family members are involved in multi-drug resistance. These energy-dependent efflux pumps are responsible for decreased drug accumulation. |
| ***ABCB4*** | ATP-binding cassette, sub-family B (MDR), member 4 |  |
| ***ABCC12*** | ATP-binding cassette, sub-family C (MRP), member 12 |  |
| ***ABCF3*** | ATP-binding cassette, sub-family F  (GCN20), member 3 |  |
| ***ABCG1*** | ATP-binding cassette, sub-family G (WHITE), member 1 |  |
| ***ABCG8*** | ATP-binding cassette, sub-family G (WHITE), member 8 |  |
| ***CFTR/***  ***ABCC7*** | cystic fibrosis transmembrane conductance regulator (ATP-binding cassette sub-family C, member 7) |  |
| ***ABCG2*** | ATP-binding cassette, sub-family G (WHITE), member 2 | Xenobiotic transporter that may play an important role in the exclusion of xenobiotics from the brain.  May be involved in brain-to-blood efflux. Appears to play a major role in the multidrug resistance phenotype of several cancer cell lines |
| ***CYP1A1*** | cytochrome P450, family 1, subfamily A, polypeptide 1 | Cytochrome P450 (CYP450) enzymes are a diverse group of catalysts that contains 57 members in humans. The cytochrome P450 proteins are monooxygenases which catalyze many reactions involved in drug metabolism and synthesis of cholesterol, steroids and other lipids. CYPs have been found in all organisms tested and are ubiquitously expressed. They are found at high levels in the liver, where they have an important role in metabolism of drugs and endogenous toxic compounds (for example bilirubin). Most CYPs can metabolize numerous substrates and this counts for their major role in drug interactions. |
| ***CYP39A1*** | cytochrome P450, family 39, subfamily A, polypeptide 1 |  |
| ***CYP26B1*** | cytochrome P450, family 26, subfamily B, polypeptide 1 |  |
| ***CYP4X1*** | cytochrome P450, family 4, subfamily X, polypeptide 1 |  |
| ***CYP4F12*** | cytochrome P450, family 4, subfamily F, polypeptide 12 |  |
| ***CA9*** | Carbonic anhydrase IX | It is a key hypoxia regulated gene known to be associated with chemoresistance. |
| ***UGT1A1*** | UDP glucuronosyltransferase 1 family, polypeptide A1 | UDPGT is of major importance in the conjugation and subsequent elimination of potentially toxic xenobiotics and endogenous compounds. |
| ***SLC17A3*** | solute carrier family 17 (sodium phosphate), member 3 | May be involved in actively transporting phosphate  into cells via Na(+) cotransport. |
| ***MGST1*** | microsomal glutathione S-transferase 1 | Conjugation of reduced glutathione to a wide number of exogenous and endogenous hydrophobic electrophiles. |
| ***HERPUD1*** | homocysteine-inducible, endoplasmic reticulum stress-inducible, ubiquitin-like domain member 1 | Plays a crucial role in ER stress resistance of cells. |
